# Prevalence of and characteristics associated with in-hospital mortality in a Ugandan neurology ward

**DOI:** 10.1186/s12883-020-1627-5

**Published:** 2020-01-31

**Authors:** Monica M. Diaz, Xin Hu, Brenda T. Fenton, Ivan Kimuli, Allison Lee, Hayley Lindsey, Jeffrey K. Bigelow, Samuel Maiser, Hamada H. Altalib, Jason J. Sico

**Affiliations:** 1 Department of Neurosciences, University of California San Diego, 220 Dickinson Street, Mail Code 8231, San Diego, CA 92103 USA; 2 University of California San Diego Health, 220 Dickinson Street, Mail Code 8231, San Diego, CA 92103 USA; 3 Johnson and Johnson Global Scholars Program, Yale School of Medicine, 20 York Street, New Haven, CT 06510 USA; 4 Yale Center for Analytical Science, Yale School of Public Health, New Haven, CT USA; 5 Chronic Disease Epidemiology, Yale School of Public Health, New Haven, CT USA; 6 Pain Research, Informatics, and Multi-morbidities, and Education (PRIME) Center, VA Connecticut Healthcare System, West Haven, CT USA; 7 Mulago Hospital and Makerere University, Kampala, Uganda; 8 Intermountain Medical Center, Salt Lake City, UT USA; 9 Departments of Neurology and Internal Medicine, University of Minnesota, Minneapolis, MN USA; 10 Hennepin Healthcare, Minneapolis, MN USA; 11 Department of Neurology, Yale University School of Medicine, New Haven, CT USA; 12 Department of Psychiatry, Yale School of Medicine, New Haven, CT USA; 13 Center for NeuroEpidemiological and Clinical Neurological Research, Yale School of Medicine, New Haven, CT USA; 14 Neurology Service, VA Connecticut Healthcare System, West Haven, CT USA; 15 Department of Internal Medicine, Yale School of Medicine, New Haven, CT USA; 16 Clinical Epidemiology Research Center (CERC), VA Connecticut Healthcare System, West Haven, CT USA

**Keywords:** Uganda, Neurological illness, Neurological infections, Stroke, Head trauma, Global neurology

## Abstract

**Background:**

While the burden of neurologic illness in developing countries is increasing, less is known about mortality among patients admitted to sub-Saharan African hospitals with neurologic disease. We sought to characterize the rate and patient-level predictors of in-hospital mortality in a Ugandan Neurology ward.cc.

**Methods:**

Data was prospectively collected on 335 patients admitted to the Neurology ward of Mulago Hospital, Kampala, Uganda. Kaplan-Meier survival curves and multivariate COX proportional hazard modeling were used to assess survival.

**Results:**

Within our sample (*n* = 307), 35.8% received no diagnosis at time of hospital admission. Stroke (27.3%), head trauma (19.6%), and malaria (16.0%) were the most common diagnoses. Among the 56 (18.5%) patients who died during the index hospitalization, the most common diagnosis at admission and at death was stroke. Adjusted regression analysis showed that patients without a diagnosis at time of death (HR = 7.01 [2.42–20.35], *p* < .001) and those with diagnoses of infections (HR = 5.21 [2.16–12.58], p = <.001), stroke (HR = 2.69 [1.20–6.04], *p* = .017), or head trauma (HR = 3.39, [1.27–9.07], *p* = 0.15) had worse survival.

**Conclusions:**

In-hospital mortality affected nearly 20% of the cohort, with worse survival among those without a diagnosis and with infections, stroke, head trauma. Future work should identify reasons for increased mortality among these high-risk groups and implement targeted interventions.

## Background

The burden of neurological illness in Africa is astounding, especially in Sub-Saharan Africa, where stroke, epilepsy, neurological complications of HIV/AIDS, systemic and central nervous system infections, and malnutrition are leading causes of mortality [1–3]. In Uganda, a country of 36 million people, non-communicable diseases (NCDs) such as stroke are estimated to account for 27% of total deaths [4]. Insufficient access to treatment, a dearth of neurologists (0.04 neurologists per 100,000 population) practicing in sub-Saharan Africa, and social stigma of neurological illnesses, including stroke and epilepsy, are potential contributors of lower access to neurological care and higher rates of mortality [1, 3, 5–8].

While there is a pronounced need to describe the prevalence of neurological illness and identify predictors of mortality associated with these diagnoses in healthcare settings throughout Uganda and other sub-Saharan countries, electronic health record (EHR) systems, and hence, administrative data, are not widely available to aid in such endeavors. Several studies have analyzed in-hospital mortality rates and associated factors in various sub-Saharan African countries, with no prior work analyzing in-hospital mortality among patients admitted to a neurology ward particularly within Uganda [9–14]. Understanding the prevalence and identifying high-risk causes of mortality is critical for healthcare providers and policy makers to prioritize public health, clinical, and quality improvement interventions. To address this gap in our understanding of care trajectories for patients hospitalized with neurological disease in sub-Saharan Africa, we longitudinally characterized the prevalence of neurological disorders and predictors of in-hospital mortality among patients admitted to the neurology ward within Mulago Hospital, the country’s largest tertiary care referral center.

## Methods

### Patient population

The study team longitudinally collected data on 335 patients admitted to the neurology ward of Mulago Hospital in Kampala, Uganda from January 2009 to May 2011. Mulago Hospital is situated within Kampala, the capital city of Uganda, a city of 1.5 million inhabitants with a high population density of 22,700 persons/m^2^ (8800 per km^2^). Much of the population of Kampala is under the age of 18 (41.3%), with 1.2% of the population being over the age of 65 [15]. The average life expectancy of citizens of Uganda during the study period was 57 years [16]. Mulago Hospital is the largest tertiary care center and the only national referral center hospital in Uganda.

### Standard protocol approvals, registrations, and patient consents

The institutional review boards of Yale School of Medicine and Makerere University approved of this study. All consent materials were available in both English and Luganda (Additional file 1: Table S1). Translators were secured for patient interactions to ensure that patients understood the consent process.

### Data collections and measures

All patients admitted to the neurology ward were eligible for enrollment into the study. Targeted interviews with patients, their attendants/family members, and when available, review of paper medical records that patients may have brought with them to the hospital were used to gather data. A standard data form formulated by US and Ugandan study physicians (JJS, IK) was used for data collection (Additional file 1: Table S2). Patients were first identified upon arrival to the neurology inpatient ward after being triaged through the Accident and Emergency ward and assigned to the neurology ward by the emergency ward provider for a suspected neurological diagnosis. Patients admitted from the emergency room to another hospital ward were not considered for enrollment. Verbal informed consent was obtained by study staff. Data collected included: demographic information, village of residence, up to five records of past medical history, admission/discharge dates and up to three admission diagnoses and discharge diagnoses assigned by the treating clinical provider, discharge disposition, and mortality data without the benefit of an EHR.

As per usual care, each patient was examined by a treating physician and focal findings on the neurologic examination were recorded in the patients’ paper medical records. For those patients who died during the index hospitalization, the cause of death noted by the treating clinical providers was recorded. Diagnoses were assigned by the treating clinical provider of the neurology ward at (1) the time of admission and (2) the time of hospital discharge or at time of death based on his or her clinical judgment and the results of diagnostic testing, when available. A diagnosis of stroke was made based on clinical suspicion and results of brain CT scan, if available. Treating providers would list a primary admission diagnosis and a primary discharge diagnosis as well as additional diagnoses he or she deemed pertinent to the presentation (e.g., a primary admission diagnosis of stroke with a secondary diagnosis of urinary tract infection). Physicians caring for patients could also not include diagnoses at admission, discharge, or both, and could include a non-neurological diagnosis as one of up to three admission or discharge diagnoses.

Patients were followed daily throughout the course of their hospitalization by the research team in order to understand patient care trajectories. Patients were not followed after hospital discharge, although the research team made note if a patient was re-hospitalized during the 29-month study period, which did not occur. Clinicians who were not participating in the clinical care of the patient abstracted data from paper patient medical records during the course of the hospitalization from the time to arrival to the hospital ward until discharge or death.

### Statistical analysis

For descriptive analyses, we described the frequency and percentage of categorical variables, and median and range of continuous variables for the whole sample by vital status (Alive vs. Dead) at discharge. Chi-square test and Wilcoxon-Mann Whitney test were used to compare the characteristics distribution between the two groups for all diagnoses and for the primary admission or primary discharge diagnosis assigned by the provider. We collected up to up to 3 admission and discharge diagnoses, and up to 5 past medical history conditions. We counted all neurological and non-neurological conditions including multiple past medical history conditions and multiple admission and discharge diagnoses. Diagnosis groups were created based on all diagnosis codes (in which one patient could fall into several diagnosis groups) and two separate analyses using exclusively the primary admission diagnosis and the primary discharge diagnosis. A detailed list of conditions/diagnoses can be found in Additional file 1: Table S2: Data Collection Tool.

Given sample size concerns using individual diagnoses, we categorized the conditions into five groups for survival analysis: stroke (both ischemic and hemorrhagic), neurotrauma (head trauma/spinal cord insult), other non-infectious conditions (i.e. hypertension, diabetes, hyperlipidemia, atrial fibrillation, myocardial infarction, liver disease, seizure, and psychiatric illness), infectious conditions (i.e. syphilis, malaria, schistosomiasis, tuberculosis, pneumonia, HIV, and Cryptococcus/Mycobacterium Avium-intracellulare [MAI]/*Pneumocystis jiroveci* pneumonia [PJP]) and not having a diagnosis by time of discharge or death. Kaplan Meier survival curves along with the log-rank test *p*-values were generated to identify potential predictors of survival. We then conducted simple COX proportional hazard regressions to assess association between each covariates and survival, and only those significant factors were selected into final multivariate COX proportional hazard regression. In our multivariate regression model, the reference group for each diagnosis group is the group of patients without that particular diagnosis of interest. For example, the reference group for “Stroke” consisted of patients without a stroke diagnosis. Proportional Hazard assumption tests were also conducted and no violation was found. Separate sensitivity analyses were conducted as: 1) patients younger than 18 years of age were excluded; 2) removed 4 patients who identified themselves as being retired; 3) using different cutoffs for length of hospital stay (i.e. up to 5 days, 10 days, and 20 days), Additional file 1: Table S5. All sensitivity analyses showed similar results to those of the original COX model. We used SAS version 9.4 (SAS Institute, Inc., Cary, NC) to conduct all analyses, using two-sided statistical tests and an alpha of 0.05.

Anonymized data not published within this article has been made publicly available and may be accessed by any qualified investigator on Mendeley Data.

## Results

A total of 335 patients admitted to the neurology ward were recruited and enrolled into the study. Of these, 24 patients had no vital status recorded, 2 patients had missing age, 2 patients had missing gender, and 5 patients had missing discharge date, thus 302 patients’ data were used for the final analyses. Median age was 47 years old (Range = 9–95) and half were women (50.7%). The average length of hospital stay was 8.4 (standard deviation [SD] = 15.9) days. More than half (61.6%) of patients’ functional status improved by discharge, but 18.5% of the cohort died during hospitalization. While nearly 30% of patients reported being hospitalized prior to the observation period (Table 1), no patients were re-admitted during the 29-month study period, such that noen of the patients enrolled in the study returned for re-admission. Results on mortality rates following discharge were not collected. The most common self-reported past medical history diagnoses were diabetes (33.4%), HIV (17.2%) and malaria (11.0%); past neurological history diagnoses included seizures/convulsions (13.1%) and stroke (10.3%; Table 1).

**Table 1:** Patient Characteristics by Mortality Status (*N* = 302)

	Alive		Dead		Total		*P*-value
	N	%	N	%	N	%	
Total N	246	81.5%	56	18.5%	302	100.0%	
Age (Median/Range)	46	[9, 95]	54	[9, 89]	47	[9, 95]	.14
Age group							.028
> =45	127	51.6%	38	67.9%	165	54.6%	
Gender							.33
Female	128	52.0%	25	44.6%	153	50.7%	
Marital Status							.18
Married	131	53.3%	24	42.9%	155	51.3%	
Single	44	17.9%	10	17.9%	54	17.9%	
Widow/Divorced	48	19.5%	17	30.4%	65	21.5%	
Occupation							.004
Employed/Farmer	77	31.3%	10	17.9%	87	28.8%	
Unemployed/Retired	23	9.3%	12	21.4%	35	11.6%	
Student/Housewife	44	17.9%	5	8.9%	49	16.2%	
Peasant	38	15.4%	14	25.0%	52	17.2%	
Missing^b^	64	26.0%	15	26.8%	79	26.2%	
Smoking Status							.22
Never	189	76.8%	36	64.3%	225	74.5%	
Currently Smokes	32	13.0%	10	17.9%	42	13.9%	
Length of Stay (Days, Mean/SD)^a^	8.8	17.2	6.6	7.6	8.4	15.9	.14
Length of Stay (Days, Median/Range)	6	[1, 249]	4	[0, 47]	6	[0, 249]	.016
Discharge Status							N/A
Functional Status Improved	186	75.6%			186	61.6%	
Functional Status Worsened	3	1.2%			3	1.0%	
Functional Status unchanged	17	6.9%			17	5.6%	
Transferred to another service	19	7.7%			19	6.3%	
Dead			56	100.0%	56	18.5%	
Left against medical advice	21	8.5%			21	7.0%	
Any Past Medical History	161	65.4%	35	62.5%	196	64.9%	.86
Past Medical History (*N* = 290)							
Stroke	24	10.0%	6	11.8%	30	10.3%	.71
Neurologic trauma							
Head Trauma	5	2.1%	2	3.9%	7	2.4%	.36
Spinal Cord Insult	1	0.4%	0	0.0%	1	0.3%	1.00
Non-communicable disease							
Atrial Fibrillation	0	0.0%	1	2.0%	1	0.3%	.18
Myocardial Infarction	1	0.4%	0	0.0%	1	0.3%	1.00
Liver Disease	0	0.0%	0	0.0%	0	0.0%	N/A
Seizure/Convulsion	34	14.2%	4	7.8%	38	13.1%	.26
Psychiatric Illness	9	3.8%	2	3.9%	11	3.8%	1.00
Communicable disease							
Syphilis	0	0.0%	0	0.0%	0	0.0%	N/A
Malaria	25	10.5%	7	13.7%	32	11.0%	.50
Schistosomiasis	0	0.0%	0	0.0%	0	0.0%	N/A
TB	8	3.3%	3	5.9%	11	3.8%	.42
Pneumonia	0	0.0%	0	0.0%	0	0.0%	N/A
HIV	42	17.6%	8	15.7%	50	17.2%	.75
Cryptococcus/MAI/PJP^c^	2	0.8%	0	0.0%	2	0.7%	1.00

^a^*SD* Standard deviation

^b^Missing group was not counted in Chi-square calculation

^c^*MAI* Mycobacterium Avium-intracellulare, *PJP Pneumocystis jiroveci* pneumonia

At the time of admission, 35.8% of the cohort was assigned no diagnosis by the treating clinical provider, but of those who were assigned an admission diagnosis, stroke (27.3%) and head trauma (19.6%) were the most common admission diagnoses (Additional file 1: Table S3). Only one-third of the cohort had a CT scan of the brain and 11.3% of those with an infectious diagnosis had a lumbar puncture (data not shown). Death occurred about equally among women and men. More than one-third of the cohort had an unknown diagnosis at the time of death. Stroke was the most common diagnosis at time of hospital discharge (about one-third of the cohort in two separate analyses utilizing all discharge diagnoses and primary discharge diagnosis separately) and the most common diagnosis assigned at time of death (half of the cohort) by the treating clinical provider. The most common non-neurological discharge diagnosis was diabetes (29.6%) (Tables 2 & 3), as patients without a neurological condition were assigned to the neurology ward by the admitting provider based on an initial clinical suspicion. There were significantly more deaths in the stroke group when analyzing only the primary discharge diagnoses (*p* = .019) (Table 2). Among those who died, about one-third had no diagnosis at the time of hospital admission, about 46% had no diagnosis at time of death, and about one-fourth had neither an admission nor discharge diagnosis. The majority of those who died had a differing admission and discharge diagnosis, and was signficiantly greater among the group who died (*p* = 0.0005) (Additional file 1: Table S4).

**Table 2:** Discharge Diagnoses by Mortality Status (N = 302)

	Alive		Dead		Total		*P*-value^b^
	N	%^a^	N	% ^a^	N	%	
No Discharge Diagnosis	80	32.5%	26	46.4%	106	35.1%	0.049
All Discharge Diagnosis (*N* = 196)							
Stroke							.26
No	113	68.1%	14	46.7%	127	64.8%	
Yes	53	31.9%	16	53.3%	69	35.2%	
Neurologic Trauma							
Head Trauma							.16
No	149	89.8%	23	76.7%	172	87.8%	
Yes	17	10.2%	7	23.3%	24	12.2%	
Spinal Cord Insult							.69
No	157	94.6%	29	96.7%	186	94.9%	
Yes	9	5.4%	1	3.3%	10	5.1%	
Non-communicable disease							
Diabetes							.51
No	117	70.5%	21	70.0%	138	70.4%	
Yes	49	29.5%	9	30.0%	58	29.6%	
Hyperlipidemia							N/A
No	166	100.0%	30	100.0%	196	100.0%	
Yes	0	0.0%	0	0.0%	0	0.0%	
Atrial Fibrillation							.31
No	162	97.6%	28	93.3%	190	96.9%	
Yes	4	2.4%	2	6.7%	6	3.1%	
Myocardial Infarction							1.00
No	165	99.4%	30	100.0%	195	99.5%	
Yes	1	0.6%	0	0.0%	1	0.5%	
Liver Disease							1.00
No	164	98.8%	30	100.0%	194	99.0%	
Yes	2	1.2%	0	0.0%	2	1.0%	
Seizure/Convulsion							.14
No	147	88.6%	29	96.7%	176	89.8%	
Yes	19	11.4%	1	3.3%	20	10.2%	
Psychiatric Illness							.13
No	139	83.7%	28	93.3%	167	85.2%	
Yes	27	16.3%	2	6.7%	29	14.8%	
Communicable disease							
Syphilis							N/A
No	166	100.0%	30	100.0%	196	100.0%	
Yes	0	0.0%	0	0.0%	0	0.0%	
Malaria							.36
No	159	95.8%	30	100.0%	189	96.4%	
Yes	7	4.2%	0	0.0%	7	3.6%	
Schistosomiasis							N/A
No	166	100.0%	30	100.0%	196	100.0%	
Yes	0	0.0%	0	0.0%	0	0.0%	
TB							1.00
No	164	98.8%	30	100.0%	194	99.0%	
Yes	2	1.2%	0	0.0%	2	1.0%	
Pneumonia							.001
No	154	92.8%	20	66.7%	174	88.8%	
Yes	12	7.2%	10	33.3%	22	11.2%	
HIV							1.00
No	154	92.8%	28	93.3%	182	92.9%	
Yes	12	7.2%	2	6.7%	14	7.1%	
Cryptococcus/MAI/PJP ^c^							N/A
No	166	100.0%	30	100.0%	196	100.0%	
Yes	0	0.0%	0	0.0%	0	0.0%	
All Discharge Diagnosis Group (*N* = 196)							
Stroke							.26
No	113	68.1%	14	46.7%	127	64.8%	
Yes	53	31.9%	16	53.3%	69	35.2%	
Neurologic Trauma							.43
No	140	84.3%	22	73.3%	162	82.7%	
Yes	26	15.7%	8	26.7%	34	17.3%	
Non-communicable Disease							.017
No	67	40.4%	17	56.7%	84	42.9%	
Yes	99	59.6%	13	43.3%	112	57.1%	
Communicable Disease							.13
No	133	80.1%	18	60.0%	151	77.0%	
Yes	33	19.9%	12	40.0%	45	23.0%	
Primary Discharge Diagnosis (*N* = 196)							
Stroke	46	27.7%	15	50.0%	61	31.1%	.019
Neurologic Trauma							
Head Trauma	12	7.2%	3	10.0%	15	7.7%	.71
Spinal Cord Insult	9	5.3%	1	3.3%	10	5.0%	1.00
Non-communicable disease							
Diabetes	28	16.9%	3	10.0%	31	15.8%	.43
Hyperlipidemia	0	0.0%	0	0.0%	0	0.0%	N/A
Atrial Fibrillation	0	0.0%	0	0.0%	0	0.0%	N/A
Myocardial Infarction	1	0.6%	0	0.0%	1	0.5%	1.00
Liver Disease	2	1.2%	0	0.0%	2	1.0%	1.00
Seizure/Convulsion	17	10.2%	1	3.3%	18	9.2%	.32
Psychiatric Illness	23	13.9%	2	6.7%	25	12.8%	.38
Communicable disease							
Syphilis	0	0.0%	0	0.0%	0	0.0%	N/A
Malaria	6	3.6%	0	0.0%	6	3.1%	.59
Schistosomiasis	0	0.0%	0	0.0%	0	0.0%	N/A
TB	2	1.2%	0	0.0%	2	1.0%	1.00
Pneumonia	6	3.6%	3	10.0%	9	4.6%	.14
HIV	11	6.6%	2	6.7%	13	6.6%	1.00
Cryptococcus/MAI/PJP ^c^	0	0.0%	0	0.0%	0	0.0%	N/A
Primary Discharge Diagnosis Group (*N* = 196)							.049
Stroke	46	27.7%	15	50.0%	61	31.1%	
Neurologic Trauma	21	12.7%	4	13.3%	25	12.8%	
Non-communicable Disease	74	44.6%	6	20.0%	80	40.8%	
Communicable Disease	25	15.1%	5	16.7%	30	15.3%	

^a^Column percent among those with non-missing discharge diagnosis

^b^Fisher Exact test was used for small cells

^c^Abbreviations: *MAI* Mycobacterium Avium-intracellulare, *PJP Pneumocystis jiroveci* pneumonia

Note: Disease groups using all discharge diagnosis codes are not mutually exclusive, and disease groups using primary discharge diagnosis codes are mutually exclusive

**Table 3:** Multivariate COX Proportional Hazard Model (*N* = 302)

	HR (95%CI)	*P*-value	PH assumption *P*-value
Age group			
< 45	Ref		
> =45	1.69 (0.88–3.25)	.12	.053
Occupation categories			
Employed/Farmer	Ref		
Unemployed/Retired	2.82 (1.16–6.86)	.022	1.00
Student/Housewife	0.80 (0.26–2.43)	.69	.63
Subsistence Farmer/Peasant	2.91 (1.25–6.78)	.013	.84
*Missing*^a^	1.82 (0.80–4.14)	.16	.79
All Discharge Diagnosis Groups			
Stroke	2.69 (1.20–6.04)	.017	.53
Head Trauma/Spinal Cord Insult	3.39 (1.27–9.07)	.015	.63
Other Non-infectious	1.24 (0.56–2.75)	.60	.58
Infectious	5.21 (2.16–12.58)	<.001	.53
No discharge diagnosis	7.01 (2.42–20.35)	<.001	.47

^a^Missing group was not counted in Chi-square calculation

**Fig. 1:**
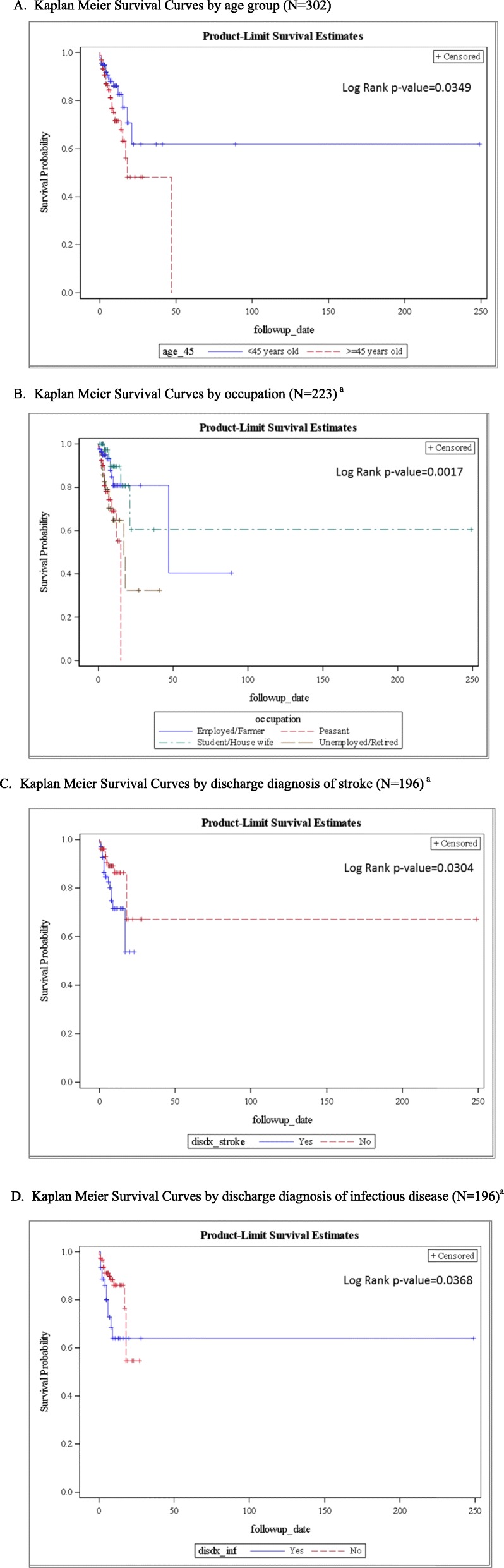
Kaplan Meier Survival Curves. A. Kaplan Meier Survival Curves by age group (*N* = 302). B. Kaplan Meier Survival Curves by occupation (*N* = 223)^a^. C. Kaplan Meier Survival Curves by discharge diagnosis of stroke (*N* = 196)^a^. D. Kaplan Meier Survival Curves by discharge diagnosis of infectious disease (*N* = 196)^a^. (B,C,D) legend: ^a^ Missing groups and patients missing follow-up time were not counted in Kaplan Meier analysis

In Kaplan Meier survival analyses, older age group was found to be significantly associated with worse survival (*p* = .0349, Fig. 1a). No statistical difference in survival was found between men and women (data not shown). In addition, unemployed/retired patients and persons in the subsistence farmer/peasant occupational groups showed significantly worse survival than employed patients/farmers (Log-rank test *p* = .0017, Fig. 1b). A diagnosis of stroke at the time of death or hospital discharge was associated with mortality (*p* = .0304, Fig. 1c), as were infectious conditions (*p* = .0368, Fig. 1d).

In our final multivariate regression model, we adjusted for occupation and discharge diagnosis. Compared to employed patients/farmers, those who were unemployed/retired (HR = 2.82, 95% CI = 1.16–6.86, *p* = .022) or subsistence farmer/peasant (HR = 2.91, 95% CI = 1.25–6.78, *p* = .013) had worse survival. A greater risk of death was seen in patients with no diagnosis at hospital discharge or time of death (HR = 7.01, 95% CI 2.42–20.35, *p* < .001) and diagnoses of stroke (HR = 2.69, 95% CI = 1.20–6.04, *p* = .017), head trauma (HR = 3.39, 95% CI = 1.27–9.07, *p* = .015; Table 3), and communicable disorders (HR = 5.21, 95% CI = 2.16–12.58, p < .001) at hospital discharge or time of death. In the sensitivity analyses using different follow-up cut-off points (up to 5 days, 10 days and 20 days follow-up, similar effect sizes were noted compared with our original multivariate regression model of entire length of hospital stay (Additional file 1: Table S5).

## Discussion

Nearly 20% of patients admitted to a Ugandan neurology ward died, with worse survival occurring among unemployed/retired persons and subsistence farmers/peasants and those with no diagnosis at time of death and a diagnosis of stroke. Among patients admitted to the neurology ward with a neurological diagnosis, there was a greater hazard of in-hospital mortality compared to admission to the same ward with a non-neurological diagnosis.

Several studies have analyzed the prevalence of neurologic illness within sub-Saharan Africa, many of which are community survey studies [9, 10, 13, 14, 17, 18]. This is the first report of the distribution of neurologic diagnoses and in-hospital mortality in a Ugandan neurology ward. Among community survey studies, one door-to-door survey study within a rural district of Uganda determined the point prevalence of neurological illness in the community was 3.3%, with peripheral neuropathy being most common (33.7%), followed by chronic headaches, stroke and epilepsy [18]. We reported a different distribution of neurological diagnoses, with differences likely attributable to the setting where sampling occurred (outpatient versus inpatient) and the geographic focus (one district in Uganda compared to every district in Uganda). The prevalence of neurological illness among hospitalized patients varies. One Nigerian inpatient study reported 24.2% of all inpatients had a neurological diagnosis [13], a study in an urban Ethiopian hospital reported a neurological diagnosis prevalence of 18% [9], and a study from Central Ghana reported 15% (of these, stroke comprised 54% and CNS infections 27%) [14]. Because our study included only patients admitted to a neurology ward, we do not report the prevalence of neurological illness amongst all patients presenting to Mulago Hospital.

Few studies in sub-Saharan Africa have reported the distribution of neurological diagnoses among inpatients with neurological illness admitted to a neurology ward and only one study reported predictors of mortality. One retrospective study from Cameroon found a similar mortality rate (19%) to that in our study with greatest mortality among those with stroke diagnoses (53%) [10]. Only one prospective study was identified in the literature reporting prevalence of neurological diagnoses in hospitalized patients in Congo, with a lower prevalence of stroke than that in our study (6.6% stroke) which may have been underestimated as there was no access to imaging [11]. Combined, these studies demonstrate that neurological illness is routinely encountered in community and inpatient settings throughout sub-Saharan Africa. Overall inpatient mortality in our study was comparable to other prevalence studies throughout sub-Saharan Africa from Ethiopia (22%) and Cameroon (19%) [9, 10], was lower than that reported by two studies from Nigeria (34%) and Central Ghana (31%) [13, 14], and was higher than one study from Congo (8.2%) [11] . Inaptient mortality due to neurological illness is variable, likely attributable to factors that differ between countries, including socioeconomic factors, healthcare systems, or data collection methods. The true mortality rate related to neurological illness in the country may have been underestimated, as data regarding patients that potentially died en route to the hospital or died in the Accident and Emergency ward prior to being hospitalized were not collected. In addition, patients were not included if they were admitted to a ward that was not the neurology ward. Therefore, patients who may have had a neurological illness and were inadvertently admitted to the medicine or another ward were unaccounted for, thus the hospital-wide prevalence of neurologic illness is unknown based on our study.

We did find that occupation was a strong predictor of poorer survival, such that patients in the lowest socioeconomic strata (i.e., subsistence farmer/peasant and unemployed/retired) had the highest in-patient mortality, compared with those who identified themselves as being employed or a farmer. Although not directly representative of socioeconomic status, there may be several factors that could be related to occupation as a surrogate for socioeconomic status that may have contributed to poor clinical outcomes, including poorer baseline health at the time of admission, longer distance to hospital if living in rural areas, poorer access to health care. This has been corroborated by other studies throughout SSA. One study in rural South Africa reported that lower socio-economic status was associated with higher HIV/AIDS, tuberculosis, and other communicable disease-associated mortality but no significant relationship between socioeconomic status and non-communicable diseases [19]. However, another study from the same region with an earlier time period 1994–2009 did show a similar, inverse relationship between socioeconomic status and mortality to that in our study [20]. Although these are plausible explanations, further work is needed to understand if these hypotheses explain this important finding.

Several other predictors of mortality were noted in our study, including an unknown admission diagnosis associated with increased mortality. Given limitations in diagnostic studies in our cohort, as in much of sub-Saharan Africa, more than a third of our cohort did not receive a diagnosis during their hospitalization. Diagnostic uncertainty is commonly encountered by health care providers in sub-Saharan Africa given the limited access to resources needed to evaluate patients. Research from several countries (South Africa [21], Mexico [22], China [23] and Tonga [24]) has identified substantial misclassification of in-hospital causes of death. While it is well-known that lacking a diagnosis leads to delay in appropriate care and subsequently increases the risk of death [25, 26], we are not familiar with other studies conducted within sub-Saharan Africa which report both the rate of unknown diagnoses during hospitalization and its influence on mortality. Though it stands to reason that interventions which provide resources necessary to conduct thorough evaluations would decrease the rate of unknown diagnoses and correspondingly improve mortality, this contention is speculative.

A diagnosis of stroke was predictive of poorer survival and was the most common diagnosis encountered in the Mulago Hospital neurology ward. The World Health Organization (WHO) reports that 85% of deaths globally attributable to neurologic conditions are due to cerebrovascular disease [2, 27, 28]. In sub-Saharan Africa, an estimated 9 to 13% of deaths are due to cardiovascular disease, including stroke [29], with yearly age-adjusted stroke rates four times higher in developing countries compared to developed countries [30]. Not surprisingly, recent work has sought to understand the true prevalence of stroke in sub-Saharan Africa. One community study in rural Uganda found stroke was one of the most common neurologic diagnoses with a prevalence of 14.3%, comparable to the worldwide stroke prevalence [18, 31]. Similarly, stroke prevalence among hospitalized patients in Ghana has increased from less than 2% in 1960 to 12% in 1993 [32]. Other studies throughout sub-Saharan Africa have found that mortality is high among stroke patients and is the leading cardiovascular cause of death and disability in sub-Saharan Africa, but further research is required to elucidate specific mortality predictors among stroke patients [33–35].

Reasons for increased mortality related to stroke may be explained by inadequate delivery of guideline concordant stroke care. For example, in the INTERSTROKE study, the mean time for completion of a CT or MRI of the brain during hospital stay was 30 h, with vascular imaging having been performed in only 2.4% of the African cohort, transthoracic echocardiography was performed in a minority of patients (10%) [36]. Similarly, a Rwandan study reported median time to hospital presentation (from time last-seen-well to emergency room presentation) was 72 h for ischemic stroke and no patients received thrombolytic therapy [37]. This is a stark contrast to the time to presentation in the U.S., usually within 6 h [38]. In our study, no patient received thrombolytic therapy as neither the medication nor stroke protocols, which included rapid assessment of acute neurologic illness with urgent CT scan, were available. No MRI imaging was available in our study, thus the suspected diagnosis of ischemic stroke may have been under-represented in our study as diagnosis of stroke was assigned by the treating clinical provider based on clinical suspicion. Resources required for inpatient stroke management are unavailable or inaccessible to most patients given out-of-pocket costs patients must pay for testing; resources for secondary stroke prevention after discharge are often also inadequate.

While we identified several important predictors of mortality, including not receiving a diagnosis and receiving a diagnosis of neurological non-communicable diseases (e.g., stroke and head trauma), being diagnosed with a non-neurological non-communicable disease (e.g., diabetes, psychiatric illness) was not associated with in-hospital mortality in this study. Many of these non-neurological non-communicable diseases tend to be chronic conditions rather than disease processes that would increase short-term mortality, which may explain why patients admitted with a neurological condition (e.g., stroke), rather than a medical condition (e.g., hypertension), had higher rates of mortality. This is contrary to reports from the WHO that report 33% of mortality (including in-hospital and outpatient mortality) is due to non-communicable diseases, but this number includes non-communicable neurological diagnoses including stroke and head trauma and does not report in-hospital mortality [4]. In addition, these non-communicable diseases may lead to acute diagnoses such as stroke, sepsis, or other inpatient cause for admission, but the non-communicable disease itself may not have been considered the primary cause of death by the treating provider in our sample. Healthcare providers practicing in sub-Saharan Africa should be aware that patients admitted with neurological conditions to have higher in-hospital mortality compared to patients admitted with more chronic medical conditions.

Limitations to our study are worth noting. First, these data are subject to the known limitations of a cross-sectional study [39]. Second, this study was conducted entirely without the benefit of an EHR. EHR or a hospital-based patient registry would have allowed for a more comprehensive assessment of neurologic disorders across the hospital rather than limited to the neurology ward, thus our data on mortality rate and predictors of mortality is not generalizable across Mulago Hospital. While administrative data would likely allow for a more complete assessment of the relationship of patient-level factors and survival within the neurology ward of Mulago Hospital, our current work provides a sound description of the mortality rate and its associated predictors, including not being assigned a diagnosis by a treating physician, within a neurology ward. Until EHRs are more widely used in Uganda and other African countries, longitudinal data collection efforts similar to our own will be required to understand mortality and other outcomes among patients hospitalized with neurological illness as well as the impact of interventions designed to mitigate mortality. Third, our data collection was restricted to Ugandans admitted to the neurology ward with a presumed disorder severe enough to require inpatient treatment, and we did not collect data on death following hospital discharge. As mentioned previously, patients who died before being admitted from the Accident and Emergency ward or were admitted to the 4-bed intensive care unit would not have been accounted for. In our experience, it was not uncommon for patients to have a 2–3 day Accident and Emergency ward stay prior to arrival on the ward. Those with suspected CNS infections were often admitted to the infectious diseases ward and may have been under-represented. We have also found that many patients admitted with road traffic accidents (e.g., those involving boda bodas) [40] and severe head trauma who were unable to receive neurosurgical intervention from either of the two neurosurgeons, who covered the entire country of Uganda during the study period, experienced poorer outcomes prior to being hospitalized [3]. Also, patients admitted with a diagnosis of seizures may have been admitted to either the neurology ward or the psychiatric ward, dependent on the disposition of the treating provider. Given these considerations, our prevalence estimates may under-report the association between head trauma, seizures, infectious diseases and more fulminant disease and mortality. Fourth, treating providers may have provided a non-neurological diagnosis for the reason for admission or discharge (e.g., hypertension). While it is our experience that treating providers may have thought that non-neurological conditions were the reason for or contributed to a patient’s presentation, we did not formally interview healthcare providers their considerations in assigning specific diagnoses, or not assigning a diagnosis. Fifth, the neurologic diagnoses accounted for in this study were assigned by the treating clinical provider often without the benefit of neuroimaging, lumbar puncture and other diagnostic modalities routinely available in developed countries, with a minority of patients without an admission or discharge diagnosis. The treating clinical provider assigned a diagnosis based on their clinical judgement and any diagnostic tests available, however, we do not have evidence that the treating provider may have used a diagnosis from the past medical history when a primary neurological diagnosis was unknown. Given diagnostic uncertainty in resource-limited settings due to inaccessibility of many diagnostic modalities, diagnoses assigned were based on the medical judgment of the treating clinical provider. When the suspected diagnosis was unknown, no diagnosis was assigned or a known diagnosis from the past medical history (such as hypertension, atrial fibrillation, diabetes) thought to be contributing to the neurological condition was assigned when the neurological diagnosis was unknown. We did not identify reasons why providers assigned certain diagnoses to patients nor why certain providers assigned the patient to the neurology ward as the diagnosis assignment was left to the local Ugandan clinical provider who routinely cares for the neurology ward patients. However, these current data identify patients at increased risk of in-hospital mortality and can be used to guide quality improvement work directed at understanding more specific reasons related to mortality among these high-risk patients. Future work could also address reasons why certain diagnoses are assigned in these resource-limited settings. In addition, the study team did not follow patients after discharge. As such, we do not have prevalence and outcomes data of outpatient neurologic illness. Brain imaging, while potentially available to patients in-hospital, was obtained in a minority of patients. This was largely due to the inability of patients to pay out-of-pocket costs for these testing. Therefore, the diagnosis of stroke was largely made on clinical grounds when patients presented with such symptoms as sudden onset of paresis/paralysis, numbness, change in speech, or vision loss [41]. Because of this limitation, we analyzed ischemic and hemorrhagic stroke together, and could not report the prevalence of each stroke type. Finally, these data are from 2009 to 2011. More recent data collection would be required to understand the current state of neurological illness and mortality and allow for analysis of trends over time.

## Conclusions

Despite these limitations, our study is one of the first to investigate the mortality rates and predictors of in-hospital morality on a neurology ward in sub-Saharan Africa. Future longitudinal work could focus investigations of health system factors that may be associated with in-hospital mortality (level of supervision and neurologic training of the clinical care team, access to an intensive care unit, healthcare worker strikes, refusal rates of lumbar puncture, availability and application of supportive care measures). By more thoroughly understanding the breadth and prevalence of neurological illness as well as predictors of poorer in-hospital survival, these data may serve to inform healthcare providers and policy makers about the development, implementation, and evaluation of interventions designed to mitigate mortality, especially among patients at high risk for poorer survival. Our findings specifically support further work on improving mortality among the Ugandans identified as being unemployed/retired persons and subsistence farmers/peasants, and those admitted with commonly encountered non-communicable (i.e., stroke, head trauma) and communicable disorders. These results also bring to light the rate and associated increased mortality of not having a diagnosis at admission or at time of death.

## Supplementary information

**Additional file 1: Table S1.** English and Luganda Consent Documentation. **Table S2.** Data Collection Tool. **Table S3.** Admission Diagnoses by Mortality Status. **Table S4.** Missingness of Admission and Discharge Diagnoses. **Table S5.** Sensitivity Analses: Lengths of Hospital Stay up to 5, 10 and 20 Days.

## Data Availability

The datasets generated and/or analyzed during the current study are available at Mendeley Data. Data can be found at the following link: Diaz, Monica; Sico, Jason (2019), “Prevalence of and Characteristics Associated with In-Hospital Mortality in a Ugandan Neurology Ward”, Mendeley Data, V2, doi: 10.17632/dg9bmcvpyp.2

## Abbreviations

AIDS: Acquired Immune Deficiency Syndrome
CI: Confidence Intervals
CNS: Central Nervous System
CT: Computerized Tomography
HER: Electronic Health Record
HIV: Human Immunodeficiency Virus
HR: Hazard Ratios
MAI: Mycobacterium Avium-intracellulare
MRI: Magnetic Resonance Imaging
NCD: Non-communicable Diseases
PJP: *Pneumocystis jiroveci* pneumonia
SD: Standard Deviation
WHO: World Health Organization
