# Supplementary material for: Prevalence of and characteristics associated with in-hospital mortality in a Ugandan neurology ward
Source: BMC Neurol. 2020 Jan 31;20:42. doi: 10.1186/s12883-020-1627-5 (PMC6995141; doi:10.1186/s12883-020-1627-5)
Supplement: Supplementary file 1 — Additional file 1: Table S1. English and Luganda Consent Documentation. Table S2. Data Collection Tool. Table S3. Admission Diagnoses by Mortality Status. Table S4. Missingness of Admission and Discharge Diagnoses. Table S5. Sensitivity Analses: Lengths of Hospital Stay up to 5, 10 and 20 Days. [file 12883_2020_1627_MOESM1_ESM.docx]

**Supplemental Material**

Table I: English and Luganda Consent Documentation

Table II: Data Collection Tool

Table III: Admission Diagnoses by Mortality Status

Table IV: Missingness of Admission and Discharge Diagnoses

Table V: Sensitivity Analyses: Lengths of Hospital Stay up to 5, 10 and 20 Days

**Table S1:** English and Luganda Consent Documentation

**Neurological Disorders Presenting to Mulago Hospital**

You are invited to participate in a project where we will be collecting data about your medical condition, including background, physical exam, and results of tests, along with diagnoses. We are collecting this data to better understand the different types of neurological cases that are seen at Mulago Hospital. This data will be used for research purposes only and will be kept confidential. Patient’s names or identifying information will not be associated with any of the finished data. Participation in this project is voluntary. If you would not like your data to be included, please let us know, and we will discard the form.

This information should help us better understand what neurological diseases are common in Uganda and will help us better treat these diseases. We thank you for your participation.

**Okulaga obulwade bwo’busuwa bwo’busimu mumubiri eri edwaliro lye’Mulago**

Oyitibwa okwetaba mu kunoonyereza kwaffe mwetujja okufuna ebikwata kumbeera yo'bujanjabi bwo ne'bogifaako emabega, nga mulimu okumanya okukeberebwa omubiri no'musaayi gwo ebirabibwa mudwaliro lye’Mulago. Bwiino ono wakukozesebwa mu byo’kunoonyereza ku bulwade buno byoka era bwiino ono ajakuba wakyaama. Amanya go’mulwade ne bwafaanana tebijja kugatibwa nebyo ebinaaba bikunganyizidwa.

Okwetaba mu kikolwa kino, kwabweereere. Bwoba nga tewandi yagadde bwiino wo okumuteekamu, tutegeeze era olupapula lwo lujja kugibwamu. Bwiino ono wa kutuyamba mu kutegeera ebika byendwade zo’busuwa bwo’busimu ezimanyikidwa mu Uganda era bwiino ono wakutuyamba mukujanjaba obulungi abalina obulwade buno.

Tukwebaza okwenyigira mu kawefube ono.

**Table S2:** Data Collection Tool

Patient Number: __________________

| **Demographic Information** | | | | | | | | | | | | | |
| --- | --- | --- | --- | --- | --- | --- | --- | --- | --- | --- | --- | --- | --- |
| [ ] VERBAL CONSENT OBTAINED  Name: District:  Age:  Gender: Village:  [ ] Male (1)  [ ] Female (2) Tribe:  Registration Number:  Married: Occupation:  [ ] Married (1)  [ ] Single (2)  [ ] Widow/widower (3/4)  [ ] Divorced (5) | | | | | | | | | | Date of Admission:  (DOA)  Date of Discharge:  (DOD)  Length of Admission (d): ______  Admitted from: (AdmitFr)  [ ] Home (1)  [ ] Mulago Clinic (2)  [ ] Outside Clinic (3)  [ ] Outside Hospital (4) | | | |
| **Condition at Discharge (DiscStatus)** | | | | | | | | | | | | | |
| [ ] **Discharged to Home** and: [ ] Transferred to another service (4)  [ ]Functional Status Improved from Admission (1) [ ] Dead, autopsy performed (5)  [ ] Functional Status Worsened from Admission (2) [ ] Dead, not autopsy performed (6)  [ ] Functional Status Unchanged from Admission (3) [ ] Left against medical advice 7 | | | | | | | | | | | | | |
| **Social History** | | | | | | | | | | | | | |
| [ ] **Smoking1:**  [ ] Yes (1) [ ] No (2) [ ] Unknown (3)  Smoking2: [ ] Yes, currently: [ ] < 10 years (1) [ ] 10-20 years (2) [ ] 21-30 years (3) [ ] 31-40 years (4) [ ] > 40 year (5)  Smoking3: [ ] Yes, previously, but quit: [ ] < 6 months ago (1) [ ] 6-12 months (2) [ ] > 12 months (3)  Packdays1: On average, packs per day: [ ] < ½ pack (1) [ ] >½ and < 1 pack (2) [ ] > 1 pack (3)  Packyears1: For how many years: [ ] < 10 years (1) [ ] 10-20 years (2) [ ] 21-30 years (3) [ ] 31-40 years (4) [ ] > 40 years (5)  [ ] **Alcohol use1:** [ ] Yes (1) [ ] No (2) [ ] Unknown (3)  Alcoholuse2: [ ] Yes, currently: [ ] daily (1) [ ] > 3 days and < 5 days/week (2) [ ] < 3 days/week (3) Alcoholuse 3: Duration: [ ] < one year (1) [ ] 1-5 years (2) [ ] 5-10 years (3) [ ] 11-20 years (4) [ ] 21-30 years (5) [ ] > 31 years (6)  Alcoholuse4: [ ] Yes, previously, but quit: [ ] < 6 months ago (1) [ ] 6-12 months (2) [ ] > 12 months (3)  Alcoholus5: Duration: [ ] < one year (1) [ ] 1-5 years (2) [ ] 5-10 years (3) [ ] 11-20 years (4) [ ] 21-30 years (5) [ ] > 31 years (6)  [ ] **Illicitdrugs1**: [ ] Yes (1) [ ] No (2) [ ] Unknown (3) | | | | | | | | | | | | | |
| **Hospital Course** | | | | | | | | | | | | | |
| **CC**:  CC1 | | | | | | | | | | | | | |
| **Admission Diagnosis**:  AdmitDx1 | | | | | | | | | | | | | |
| **Discharge Diagnosis**:  **Principal**: DDxa1  **Secondary**: DDxb1 | | | | | | | | | | | | | |
| **History of Hospitalizations**  HOH1 | | | | | | | | | | | | | |
| [ ] Index admission (1) [ ] In last 6 months Dx: _______________________ (4)  [ ] In last month Dx:___________________________ (2) [ ] In last 12 month Dx:________________________ (5)  [ ] In last 3 months Dx: ________________________ (3) [ ] In greater than 12 months Dx: ________________ (6) | | | | | | | | | | | | | |
| **Past Medical History (Known Diagnoses prior to Admission)**  PMHx1 | | | | | | | | | | | | | |
| [ ] None (0) [ ] HTN [ ] untreated (2) [ ] treated (3) [ ] DM2 [ ] untreated (4) [ ] treated (5)  [ ] Hyperlipidemia (5) [ ] Atrial fibrillation [ ] on Coumadin (6) [ ] not on (7)  [ ] Myocardial infarction (8) [ ] Liver disease [ ] With Cirrhosis (9) [ ] Without cirrhosis (10)  NEUROLOGICAL: [ ] Stroke [ ] Ischemic (11) [ ] Hemorrhagic (12)  [ ] Spinal cord insult (13) [ ] Seizure/convulsion (14) [ ] Head Trauma (15) [ ] Psychiatric illness (16) | | | | | | | INFECTIONS:  [ ] Syphilis (17) [ ] Malaria (18) [ ] Cerebral malaria (19)  [ ] Schistosomiasis (20) [ ] TB history: [ ] Yes (21) [ ] No (22)   [ ] Diagnosed when: ____________ (TBHx1)  [ ] Site of Infection: _____________ (TBHx2) [ ] Pneumonia (23)  [ ] **HIV**: [ ] Yes (24) [ ] No (25)   [ ] Diagnosed when: ____________ (HIVHx1)  [ ] Site of Infection: _____________ (HIVHx2)  [ ] Cryptococcus history: [ ] Yes (26) [ ] No (27)   [ ] MAI history: [ ] Yes (28) [ ] No (29)  [ ] *Pneumocystis jurevecii* history: [ ] Yes (30) [ ] No (31)  If yes, details:_______________________ | | | | | | |
| **Medications** | | | | | | | | | | | | | |
| **Home Medications** (HomeMeds1) [ ] None (1) [ ] Never taken prescription medication prior to index admission (2)  [ ] Prescribed medication: [ ] is taking (3) [ ] is not taking (4)   [ ] Herbals: (5) | | | | | | | | | | | | | |
| **Hospital Medications** (HospitalMeds1)  [ ] Antibiotics (1) [ ] Antimalaria (2) [ ] Aspirin (3) [ ] ARV (4) [ ] Blood pressure med (5) [ ] Cholesterol med (6)  [ ] Coumadin (7) [ ] Seizure medication (8) [ ] Steroids (9) [ ] TB Med (10)  [ ] Other: _________________________________________ (11) | | | | | | | | | | | | | |
| **Discharge Medications** (DischargeMed1)  [ ] Antibiotics (1) [ ] Antimalaria (2) [ ] Aspirin (3) [ ] ARV (4) [ ] Blood pressure med (5) [ ] Cholesterol med (6)  [ ] Coumadin (7) [ ] Seizure medication (8) [ ] Steroids (9) [ ] TB Med (10)  [ ] Other: _________________________________________ (11) | | | | | | | | | | | | | |
| **ROS:**  (ROS1) | | | | | | | | | | | | | |
| [ ] HA (1) | | | | | | | [ ] Urinary retention (21) [ ] Urinary incontinence (22) | | | | | | |
| [ ] Fevers/Chills (2) | | | | | | | [ ] Chest pain (23) [ ] Shortness of breath (24) | | | | | | |
| [ ] Change in vision (3) | | | | | | | [ ] Cough [ ] productive (25) [ ] non-productive (26) | | | | | | |
| [ ] Change in mental status (4) [ ] Loss of consciousness (5) | | | | | | | [ ] Hemoptysis (27) [ ] Hematemesis (28) | | | | | | |
| [ ] Unilateral weakness (6) | | | | | | | [ ] Hematochezia (29) [ ] Melena (30) | | | | | | |
| [ ] Lower extremity weakness (7) | | | | | | | [ ] Night sweats (31) [ ] other B-cell Symptoms (32) | | | | | | |
| [ ] Focal weakness (8) | | | | | | | [ ] Convulsions (33) | | | | | | |
| [ ] Numbness (9) [ ] Tingling (10) | | | | | | | [ ] Abdominal pain (34) | | | | | | |
| [ ] Neck Stiffness (11) | | | | | | | [ ] Abdominal distension (35) | | | | | | |
| [ ] Word finding difficulties (12) | | | | | | | [ ] Back pain (36) | | | | | | |
| [ ] Dysarthria (13) [ ] Diplopia (14) [ ] Dysphagia (15) | | | | | | | [ ] Radicular pain (37) | | | | | | |
| [ ] Difficulty walking (16) [ ] Falls (17) | | | | | | | [ ] Rash (38) | | | | | | |
| [ ] Constipation(18) [ ] Diarrhea (19) [ ] Incontinence (20) | | | | | | | [ ] Other (39) | | | | | | |
| **Physical Examination:** | | | | | | | | | | | | | |
| Vitals:  (Temp1): ______ (SBP1/DBP1): ______ (Pulse1): ______ (PulseOx1): ______ (RR1): ______ | | | | | | | | | | | | | |
| General Examination: (GenExam1)  [ ] NAD (1) [ ] mild distress (2) [ ] moderate distress (3) [ ] severe distress (4) [ ] well-nourished (5)  [ ] cachectic (6) | | | | | | | | | | | | | |
| HEENT/Neck: (HNeck1) [ ] normal (1) [ ] Nuchal rigidity (2) [ ] LAD (3) [ ] conjunctival lesion (4) | | | | | | | | | | | | | |
| CV: (CV1) [ ] normal (1) [ ] SEM (2) [ ] HSM (3) [ ] Diastolic murmur (4) [ ] IRR (5) [ ] S4 (6)  [ ] S3 (7) [ ] gallop (8) [ ] rub (9) | | | | | | | | | | | | | |
| Lungs: (Lungs1) [ ] normal (10) [ ] decreased on right (11) [ ] decreased on left (12) [ ] rhonchi (13)  [ ] crackles (14) | | | | | | | | | | | | | |
| Thorax: (Thorax1) [ ] Normal (15) [ ] LAD (16) | | | | | | | | | | | | | |
| Abdomen: (Abd1) [ ] Normal (17) [ ] Hepato/Splenomegaly (18) [ ] Tenderness (19) | | | | | | | | | | | | | |
| Extremity: (Extr1) [ ] Normal (20) [ ] clubbing (21) [ ] cyanosis (22) [ ] edema (23) [ ] Beau Lines (24) | | | | | | | | | | | | | |
| Skin: (Skin1) [ ] Normal (25) [ ] Rashes (26) | | | | | | | | | | | | | |
| Lymph: (Lymph1) [ ] Normal (27) [ ] LAD (28) | | | | | | | | | | | | | |
| Musculoskeletal: (Muscle1) [ ] Normal (29) [ ] Muscle tenderness (30) [ ] Spinous tenderness (31) | | | | | | | | | | | | | |
| **Neurological Examination:** (NeuroExam1) | | | | | | | | | | | | | |
| **Mental Status**: | | | | | | | | | | | | | |
| *Level of Awareness: (*Aware1)   [ ] awake (1) [ ] tired (2) [ ] lethargic (3) [ ] stupor (4) [ ] coma (5) | | | | | | | | | | | Glasgow Coma Score (GCS1): | | |
| *Language:* (Speak) | | | | | | | | | | | | | |
| *Fluency intact: Naming intact: Repetition intact: Comprehension intact:*  *(Speak1) (Speak2) (Speak3) (Speak4)* | | | | | | | | | | | | | |
| [ ] Yes (1) [ ] Yes (1) [ ] Yes (1) [ ] Yes (1)  [ ] No (2) [ ] No (2) [ ] No (2) [ ] No (2)  [ ] Not tested (3) [ ] Not tested (3) [ ] Not tested (3) [ ] Not tested (3) | | | | | | | | | | | | | |
| **CN**: CranialNerv1  [ ] normal (1)  [ ] abnormal [ ] Impaired EOM (2) [ ] Impaired pupils (2) [ ] central 7^th^ lesion (3) [ ] peripheral 7^th^ lesion (4)  [ ] impaired gag (5) [ ] difficulty with secretions (6) | | | | | | | | | | | | | |
| **Motor/Reflexes**: | | | | | | | | | | | | | |
| Is Pattern of loss present: Motor1  [ ] hemiparesis [ ] face/arm > leg (1) [ ] face/arm/leg equal (2) [ ] quadraparesis (3) [ ] paraperesis (4)  [ ] None present (5) | | | | | | | | | | | | | |
|  | *Power*  *(Motor2)* | | *Tone*  *(Motor3)* | | *Fasciculations*  *(Motor4)* | | | | *Atrophy*  *(Motor5)* | | | *Reflexes with Babinski*  *(Motor6)* | |
| RUE | [ ] Normal (1)  [ ] Increased (2)  [ ] Decreased (3)  [ ] Not recorded (4) | | [ ] Normal (1)  [ ] Increased (2)  [ ] Decreased (3)  [ ] Not recorded (4) | | [ ] Present (1)  [ ] Absent (2)  [ ] Not recorded (3) | | | | [ ] Present (1)  [ ] Absent (2)  [ ] Not recorded (3) | | | [ ] Normal (1)  [ ] Increased unilaterally (2)  [ ] Increased in lower extremities (3)  [ ] Decreased unilaterally (4)  [ ] Decreased in lower extremities (5)  [ ] Babinski positive unilaterally (6)  [ ] Babinski positive bilaterally (7) | |
| LUE | [ ] Normal (5)  [ ] Increased (6)  [ ] Decreased (7)  [ ] Not recorded (8) | | [ ] Normal (5)  [ ] Increased (6)  [ ] Decreased (7)  [ ] Not recorded (8) | | [ ] Present (4)  [ ] Absent (5)  [ ] Not recorded (6) | | | | [ ] Present (1)  [ ] Absent (2)  [ ] Not recorded (3) | | | [ ] Normal (1)  [ ] Increased unilaterally (2)  [ ] Increased in lower extremities (3)  [ ] Decreased unilaterally (4)  [ ] Decreased in lower extremities (5)  [ ] Babinski positive unilaterally (6)  [ ] Babinski positive bilaterally (7) | |
| RLE | [ ] Normal (9)  [ ] Increased (10)  [ ] Decreased (11)  [ ] Not recorded (12) | | [ ] Normal (9)  [ ] Increased (10)  [ ] Decreased (11)  [ ] Not recorded (12) | | [ ] Present (7)  [ ] Absent (8)  [ ] Not recorded (9) | | | | [ ] Present (1)  [ ] Absent (2)  [ ] Not recorded (3) | | | [ ] Normal (1)  [ ] Increased unilaterally (2)  [ ] Increased in lower extremities (3)  [ ] Decreased unilaterally (4)  [ ] Decreased in lower extremities (5)  [ ] Babinski positive unilaterally (6)  [ ] Babinski positive bilaterally (7) | |
| LLE | [ ] Normal (13)  [ ] Increased (14)  [ ] Decreased (15)  [ ] Not recorded (16) | | [ ] Normal (13)  [ ] Increased (14)  [ ] Decreased (15)  [ ] Not recorded (16) | | [ ] Present (10)  [ ] Absent (11)  [ ] Not recorded (12) | | | | [ ] Present (1)  [ ] Absent (2)  [ ] Not recorded (3) | | | [ ] Normal (1)  [ ] Increased unilaterally (2)  [ ] Increased in lower extremities (3)  [ ] Decreased unilaterally (4)  [ ] Decreased in lower extremities (5)  [ ] Babinski positive unilaterally (6)  [ ] Babinski positive bilaterally (7) | |
| **Neurological Exam (continued):** | | | | | | | | | | | | | |
| **Sensory:** | | | | | | | | | | | | | |
|  | *Light touch*  *(Sensory1)* | *Pinprick*  *(Sensory2)* | | *Temperature*  *(Sensory3)* | | | | *Vibration*  *(Sensory4)* | | *Proprioception & Romberg*  *(Sensory5)* | | | *Extinction*  *(Sensory6)* |
| RUE | [ ] Normal (1)  [ ] Abnormal (2)  [ ] Not recorded (3) | [ ] Normal (1)  [ ] Abnormal (2)  [ ] Not recorded (3) | | [ ] Normal (1)  [ ] Abnormal (2)  [ ] Not recorded (3) | | | | [ ] Normal (1)  [ ] Abnormal (2)  [ ] Not recorded (3) | | [ ] Normal (1)  [ ] Abnormal (2)  [ ] Not recorded (3) | | | [ ] Present (1)  [ ] Absent (2)  [ ] Not recorded (3) |
| LUE | [ ] Normal (4)  [ ] Abnormal (5)  [ ] Not recorded (6) | [ ] Normal (4)  [ ] Abnormal (5)  [ ] Not recorded (6) | | [ ] Normal (4)  [ ] Abnormal (5)  [ ] Not recorded (6) | | | | [ ] Normal (4)  [ ] Abnormal (5)  [ ] Not recorded (6) | | [ ] Normal (4)  [ ] Abnormal (5)  [ ] Not recorded (6) | | | [ ] Present (4)  [ ] Absent (5)  [ ] Not recorded (6) |
| RLE | [ ] Normal (7)  [ ] Abnormal (8)  [ ] Not recorded (9) | [ ] Normal (7)  [ ] Abnormal (8)  [ ] Not recorded (9) | | [ ] Normal (7)  [ ] Abnormal (8)  [ ] Not recorded (9) | | | | [ ] Normal (7)  [ ] Abnormal (8)  [ ] Not recorded (9) | | [ ] Normal (7)  [ ] Abnormal (8)  [ ] Not recorded (9) | | | [ ] Present (7)  [ ] Absent (8)  [ ] Not recorded (9) |
| LLE | [ ] Normal (10)  [ ] Abnormal (11)  [ ] Not recorded (12) | [ ] Normal (10)  [ ] Abnormal (11)  [ ] Not recorded (12) | | [ ] Normal (10)  [ ] Abnormal (11)  [ ] Not recorded (12) | | | | [ ] Normal (10)  [ ] Abnormal (11)  [ ] Not recorded (12) | | [ ] Normal (10)  [ ] Abnormal (11)  [ ] Not recorded (12) | | | [ ] Present (10)  [ ] Absent (11)  [ ] Not recorded (12) |
| **Cerebellar:** *Cerebellum1* | | | | | | | | | | | | | |
| RUE [ ] Normal (1) [ ] Abnormal (2) [ ] Not recorded (3)  LUE [ ] Normal (4) [ ] Abnormal (5) [ ] Not recorded (6) | | | | | | | | | | | | | |
| RLE [ ] Normal (7) [ ] Abnormal (8) [ ] Not recorded (9)  LLE [ ] Normal (10) [ ] Abnormal (11) [ ] Not recorded (12) | | | | | | | | | | | | | |
| Trunk [ ] Normal (9) [ ] Abnormal (10) | | | | | | | | | | | | | |
| **Gait**: *Cerebellum2*  [ ] Normal (1) [ ] Abnormal (2) [ ] Not recorded (3) | | | | | | | | | | | | | |
| **Ambulatory assist device**: AmbDev1  [ ] used (1) [ ] not used (2) [ ] Not recorded (3) | | | | | | | | | | | | | |
| **Investigations: [ ] ND = not done** | | | | | | | | | | | | | |
| Laboratory Data | | | | | | Imaging | | | | | | | |
| [ ] CBC:  [ ] Peripheral smear: [ ] malaria (1) [ ] no malaria (2)  [ ] NR  [ ] Renal Function Tests:  [ ] LFT:  [ ] Serostatus: [ ] HIV + (1) [ ] HIV – (2)  CD4 count:  [ ] CSF Analysis [ ] Done (1) [ ] Not done (2)  Cell count: _____   Protein: _____ glucose: ____  Pressure elevated: [ ]Yes (1) [ ]No (2) [ ]NR (3)  Appearance: [ ] bloody (1) [ ] yellow (2) [ ] clear (3) [ ] NR (4)  Xanthochromia: [ ] Yes (1) [ ] No (2) [ ] NR (3)  India Ink Positive:[ ] Yes (1) [ ] No (2) [ ] NR (3)  Culture positive:[ ] Yes (1) [ ] No (2) [ ] NR (3)  Gram Stain positive:[ ]Yes (1) [ ]No (2) [ ]NR (3)  [ ] Other: | | | | | | **[ ] CXR**: [ ] Positive (1) [ ] Negative (2) [ ] Unable to afford (3) [ ] NR (4) [ ] Results: ______________________________________  **[ ] Head CT:** [ ] Positive (1) [ ] Negative (2) [ ] Unable to afford (3) [ ] NR (4) Results: ______________________________________  **[ ] ECHO (echo1):** [ ] Positive (1) [ ] Negative (2) [ ] Unable to afford (3) [ ] NR4 (4)  EF (echo2): [ ] less than 20% (1) [ ] >20 to <40% (2)  [ ] > 40 (3)  Local wall motion abnormality (echo3):  [ ] Yes (1) [ ] No (2)  Valvular Heart disease (echo4): [ ] Yes (1)  [ ] No (2) __________  EMF (echo5): [ ] Yes (1) [ ] No (2)  [ ] **EKG1**: [ ] Positive (1) [ ] Negative (2) [ ] Unable to afford (3) NR (4) [ ] **EKG2**: Normal Sinus Rhythm: [ ] Yes (1) [ ] No (2) [ ] NR (3) [ ] **EKG3**: [ ] MI (1) [ ] Arrhythmia (2) [ ] Other (3) [ ] NR (4)  [ ] **EEG1:** [ ] Positive (1) [ ] Negative (2) [ ] Unable to afford (3) NR (4)  Results:______________________________________  [ ] Other: | | | | | | | |

**Table S3:** Admission Diagnoses by Mortality Status

|  | **Alive** | | **Dead** | | **Total** | | **P-value** |
| --- | --- | --- | --- | --- | --- | --- | --- |
|  | **N** | **%** | **N** | **%** | **N** | **%** |  |
| **All Admission Diagnosis (N=194)** | | | | | | | |
| Hypertension | 5 | 3.2% | 1 | 2.8% | 6 | 3.1% | 1.00 |
| Diabetes | 35 | 22.2% | 7 | 19.4% | 42 | 21.6% | .74 |
| Hyperlipidemia | 5 | 3.2% | 0 | 0.0% | 5 | 2.6% | .59 |
| Atrial Fibrillation | 3 | 1.9% | 0 | 0.0% | 3 | 1.5% | 1.00 |
| Myocardial Infarction | 0 | 0.0% | 0 | 0.0% | 0 | 0.0% | N/A |
| Liver Disease | 3 | 1.9% | 0 | 0.0% | 3 | 1.5% | 1.00 |
| Stroke | 0 | 0.0% | 0 | 0.0% | 0 | 0.0% | N/A |
| Spinal Cord Insult | 0 | 0.0% | 0 | 0.0% | 0 | 0.0% | N/A |
| Seizure/Convulsion | 42 | 26.6% | 11 | 30.6% | 53 | 27.3% | .65 |
| Head Trauma | 6 | 3.8% | 1 | 2.8% | 7 | 3.6% | 1.00 |
| Psychiatric Illness | 23 | 14.6% | 4 | 11.1% | 27 | 13.9% | .80 |
| Syphilis | 31 | 19.6% | 7 | 19.4% | 38 | 19.6% | .98 |
| Malaria | 18 | 11.4% | 2 | 5.6% | 20 | 10.3% | .55 |
| Schistosomiasis | 0 | 0.0% | 0 | 0.0% | 0 | 0.0% | N/A |
| TB | 24 | 15.2% | 7 | 19.4% | 31 | 16.0% | .54 |
| Pneumonia | 0 | 0.0% | 0 | 0.0% | 0 | 0.0% | N/A |
| HIV | 2 | 1.3% | 0 | 0.0% | 2 | 1.0% | 1.00 |
| Cryptococcus/MAI/PJP | 9 | 5.7% | 5 | 13.9% | 14 | 7.2% | .091 |
| **All Admission Diagnosis Disease Group (N=194)** | | | | | | | |
| Stroke | 84 | 53.2% | 13 | 36.1% | 97 | 50.0% | .11 |
| Head Trauma | 40 | 25.3% | 13 | 36.1% | 53 | 27.3% | .22 |
| Other Non-infectious | 42 | 26.6% | 11 | 30.6% | 53 | 27.3% | .65 |
| Infectious | 37 | 23.4% | 8 | 22.2% | 45 | 23.2% | .89 |

| **Table S4:** Missingness of Admission and Discharge Diagnoses | | | | |  | |  |
| --- | --- | --- | --- | --- | --- | --- | --- |
|  | **Alive** | | **Dead** | | **Total** | | **P-value** |
|  | **N** | **%** | **N** | **%** | **N** | **%** |  |
| **No Admission Diagnosis** |  |  |  |  |  |  | .99 |
|  | 88 | 35.8% | 20 | 35.7% | 108 | 35.8% |  |
| **No Discharge Diagnosis** |  |  |  |  |  |  | .049 |
|  | 80 | 32.5% | 26 | 46.4% | 106 | 35.1% |  |
| **No Admission nor Discharge Diagnoses** |  |  |  |  |  |  | .84 |
|  | 54 | 22.0% | 13 | 23.2% | 67 | 22.2% |  |
| **Differing admission and discharge diagnoses**  *(Non-missing primary admission dx and primary discharge dx, N=155)** | | | | | | | .005 |
|  | 74 | 56.1% | 20 | 87.0% | 94 | 60.6% |  |

* Primary admission/discharge diagnosis is the first non-missing admission/discharge diagnosis.

**Table S5:** Sensitivity Analses: Lengths of Hospital Stay up to 5, 10 and 20 Days

|  | **Entire Cohort up to 5 Days Length of Stay (N=302)** | | |
| --- | --- | --- | --- |
|  | **HR (95%CI)** | **P-value** | **PH assumption P-value** |
| **Age group** |  |  |  |
| <45 | Ref |  |  |
| >=45 | 1.04 (0.44-2.47) | .92 | .37 |
| **Occupation categories** |  |  |  |
| Employed/Farmer | Ref |  |  |
| Unemployed/Retired | 3.87 (1.07-13.95) | .039* | .74 |
| Student/Housewife | 0.47 (0.05-4.26) | .50 | .37 |
| Subsistence Farmer/Peasant | 3.94 (1.21-12.84) | .023* | .74 |
| **All Discharge Diagnosis Groups** |  |  |  |
| Stroke | 2.35 (0.86-6.43) | .098 | .21 |
| Head Trauma/Spinal Cord Insult | 2.54 (0.77-8.34) | .13 | .47 |
| Other Non-infectious | 1.07 (0.40-2.84) | .90 | .26 |
| Infectious | 3.67 (1.26-10.64) | .016 | .37 |
| No Discharge Diagnosis | 3.32 (0.90-12.27) | .072 | .42 |

|  | **Entire Cohort up to 10 Days Length of Stay (N=302)** | | |
| --- | --- | --- | --- |
|  | **HR (95%CI)** | **P-value** | **PH assumption P-value** |
| **Age group** |  |  |  |
| <45 | Ref |  |  |
| >=45 | 1.69 (0.82-3.50) | .16 | .16 |
| **Occupation categories** |  |  |  |
| Employed/Farmer | Ref |  |  |
| Unemployed/Retired | 2.37 (0.92-6.14) | .074 | .95 |
| Student/Housewife | 0.67 (0.18-2.51) | .55 | .32 |
| Subsistence Farmer/Peasant | 2.17 (0.90-5.23) | .084 | .63 |
| **All Discharge Diagnosis Groups** |  |  |  |
| Stroke | 2.84 (1.20-6.70) | .017* | .53 |
| Head Trauma/Spinal Cord Insult | 3.42 (1.19-9.81) | .022 | .63 |
| Other Non-infectious | 1.15 (0.50-2.63) | .74 | .53 |
| Infectious | 6.26 (2.51-15.61) | <.001* | .53 |
| No Discharge Diagnosis | 6.86 (2.15-21.94) | .001 | .68 |

|  | **Entire Cohort up to 20 Days Length of Stay (N=302)** | | |
| --- | --- | --- | --- |
|  | **HR (95%CI)** | **P-value** | **PH assumption P-value** |
| **Age group** |  |  |  |
| <45 | Ref |  |  |
| >=45 | 1.63 (0.84-3.17) | .15 | .37 |
| **Occupation categories** |  |  |  |
| Employed/Farmer | Ref |  |  |
| Unemployed/Retired | 2.88 (1.17-7.09) | .021 | .95 |
| Student/Housewife | 0.67 (0.20-2.25) | .51 | .63 |
| Subsistence Farmer/Peasant | 2.91 (1.24-6.83) | .014 | .79 |
| **All Discharge Diagnosis Groups** |  |  |  |
| Stroke | 2.65 (1.18-5.97) | .019* | .53 |
| Head Trauma/Spinal Cord Insult | 3.23 (1.21-8.63) | .019 | .68 |
| Other Non-infectious | 1.21 (0.55-2.68) | .64 | .58 |
| Infectious | 5.20 (2.16-12.51) | <.001* | .47 |
| No Discharge Diagnosis | 6.48 (2.22-18.90) | .0006* | .53 |
